# Adherence to and factors associated with self-care behaviours in type 2 diabetes patients in Ghana

**DOI:** 10.1186/s12902-017-0169-3

**Published:** 2017-03-24

**Authors:** Victor Mogre, Zakaria Osman Abanga, Flora Tzelepis, Natalie A. Johnson, Christine Paul

**Affiliations:** 1 Department of Health Professions Education, School of Medicine and Health Sciences, University for Development Studies, P. O. Box TL 1883, Tamale, Ghana; 2 Department of Community Nutrition, School of Allied Health Sciences, University for Development Studies, Tamale, Ghana; 3 School of Medicine and Public Health, University of Newcastle, University Drive, Callaghan, NSW 2308 Australia; 4 Hunter Medical Research Institute, Locked bag 1000, New Lambton, NSW 2305 Australia; 5 Hunter New England Population Health, Hunter New England Local Health District, Locked Mail Bag 10, Wallsend, NSW 2287 Australia

**Keywords:** Adherence, Self-care behaviours, Diabetes, Body weight, Ghana

## Abstract

**Background:**

Previous research has failed to examine more than one self-care behaviour in type 2 diabetes patients in Ghana. The purpose of this study is to investigate adult Ghanaian type 2 diabetes patients’ adherence to four self-care activities: diet (general and specific), exercise, self-monitoring of blood glucose (SMBG) and foot care.

**Methods:**

Consenting type 2 diabetes patients attending diabetes outpatient clinic appointments at three hospitals in the Tamale Metropolis of Ghana completed a cross-sectional survey comprising the Summary of Diabetes Self-Care Activities Measure, and questions about demographic characteristics and diabetes history. Height and weight were also measured. Multiple linear regression analyses were conducted to identify the factors associated with adherence to each of the four self-care behaviours.

**Results:**

In the last 7 days, participants exercised for a mean (SD) of 4.78 (2.09) days and followed diet, foot care and SMBG for a mean (SD) of 4.40 (1.52), 2.86 (2.16) and 2.15 (0.65) days, respectively. More education was associated with a higher frequency of reported participation in exercise (*r* = 0.168, *p* = 0.022), following a healthy diet (*r* = 0.223, *p* = 0.002) and foot care (*r* = 0.153, *p* = 0.037) in the last 7 days. Males reported performing SMBG (*r* = 0.198, *p* = 0.007) more frequently than their female counterparts.

**Conclusion:**

Adherence to diet, SMBG and checking of feet were relatively low. People with low education and women may need additional support to improve adherence to self-care behaviours in this type 2 diabetes population.

## Background

Diabetes has emerged as an important non-communicable disease in Sub-Saharan Africa [1]. According to the International Diabetes Federation, about 50% of all deaths attributed to diabetes were in less-developed regions like Sub-Saharan Africa [2]. Over three-quarters of these deaths occurred in individuals under 60 years old, affecting the productive work force of the sub-region. The prevalence of diabetes in adults aged 20–79 years in Ghana has increased from a prevalence of 0.2% in 1958 [3] to an estimated prevalence of 3.3% in 2014 [4]. Similar to other parts of the world, type 2 diabetes is the most common form of diabetes in Ghana [1].

The recommended self-care regimen for type 2 diabetes patients generally includes regular physical activity, healthy eating and foot care as well as self-monitoring of blood glucose (SMBG) [5, 6]. Adherence to these self-care behaviours improves glycaemic control [7]; sustains blood pressure [8]; reduces the severity of complications [7] and health costs [9].

Consistent implementation of recommended self-care behaviours for individuals with type 2 diabetes requires collaboration between the patient and the provider in an enabling health care system with adequate facilities and resources [10]. This is a major challenge for many sub-Saharan countries in the wake of the rising prevalence of diabetes [1, 11, 12] because sub-Saharan Africa is faced with inadequate facilities/resources, inadequately skilled staff, and lack of resources for diabetes education [11–13, 1].

There is, however, limited data regarding the frequency of adherence to self-care behaviours in individuals with type 2 diabetes in the sub-Saharan region including Ghana. The few studies in Ghana and other parts of the region suggests that diabetes patients adherence to self-care behaviours is low [14–18]. Ayele et al [14] reported self-care behaviour adherence of 39.2% in a sample of type 2 diabetes in Ethiopia. A cross-sectional study among a sample of type 2 diabetes patients in Nigeria found 67.4% reporting complete adherence to dietary treatment regimens [18].

A number of factors have been shown to be associated with adherence to self-care behaviours. Previous research has found an association between self-care behaviours and patients’ demographic characteristics such as age, gender, and education; doctor-patient relationships; psychological stress; and social support/context [19, 20]. Most of these studies were conducted in western countries. Our understanding of how patient demographic characteristics may be associated with self-care behaviours in type 2 diabetes in the sub-Saharan African settings is limited.

One other factor that could also influence adherence to self-care behaviours in individuals with type 2 diabetes is excess weight. Overweight and obesity are common in individuals with type 2 diabetes [21, 22]. However, only one study [23] has evaluated the association between self-care behaviours and body mass index (BMI) and waist circumference (WC) in type 2 diabetes patients in which those with BMI ≥35 Kg/m^2^ compared to those with BMI < 35 Kg/m^2^ were less likely to achieve healthy diet and exercise targets. Consequently, there is limited data regarding the influence of body weight on adherence to self-care behaviours in type 2 diabetes. The aims of this study are to describe:

1. Ghanaian type 2 diabetes patients’ adherence to the following self-care behaviours: diet, exercise, SMBG and foot care.
2. The association between adherence to self-care behaviours and patients’ demographic characteristics (including age, gender, education, and religion)
3. The association between adherence to self-care behaviours and body weight measured by body-mass index (BMI) and waist circumference.

## Methods

### Participants and setting

Participants in this cross-sectional study were type 2 diabetes patients seeking care from the out-patient diabetes clinics of the Tamale Teaching Hospital, Tamale West and Central Hospitals located in the Tamale Metropolis of Ghana. These hospitals have weekly diabetes clinics to provide care to diabetes patients. Tamale is located approximately 500 km North of Accra, Ghana’s capital. It is the administrative capital of the Northern Region of Ghana and inhabited by people of both urban and rural backgrounds.

Patients were eligible to participate if they: had a confirmed diagnosis of type 2 diabetes; self-reported healthcare professional diagnosis of type 2 diabetes; and sought care from the diabetes clinic at least twice during the last 12 months and were registered with the specific hospital. Patients were excluded if they: had type 1 diabetes; were younger than 18 years and/or were diagnosed with diabetes before the age of 30 years.

### Procedures

Letters were written to the heads of the diabetes clinics through the heads of the hospitals to seek permission for the study to be conducted on the premises. From May to June, 2015, trained research assistants visited the outpatient diabetes clinic weekly on days scheduled by the hospitals for the purposes of providing care to out-patient diabetes patients to recruit patients for the study. The research assistants approached patients while they waited for their medical consultation or after their consultation, to introduce the study to them and seek their consent to participate. Participants who agreed to participate were taken through the consent processes and subsequently given a survey to complete. Participants were advised that participation in the study was voluntary. The survey was paper-based and was self-administered to participants who could read and write in English. For those who could not read nor write in English, trained research assistants assisted them to complete the survey by translating the questions into their respective local dialects. This was observed in less than 10% of the participants. The survey took approximately 20 min to complete.

Weight, height and waist circumference were also measured by the trained research assistants after participants had completed the survey. These measurements were conducted in a secluded room at the hospital. The research was approved by the research department of the Tamale Teaching Hospital, the Ethics Committee of the School of Allied Health Sciences of the University for Development Studies and the Human Research Ethics Committee of the University of Newcastle.

### Measures

*Self-care behaviours:* The revised version of the Summary of Diabetes Self-Care Activities (SDSCA) questionnaire [24] was used to measure participants’ self-reported frequency of adhering to self-care behaviours. The SDSCA assesses participants’ frequency of engaging in diabetes self-care behaviours such as following a general diet (i.e. following healthy eating plan) and a specific diet (i.e. consuming fruits and vegetables and reducing the consumption of high fatty foods); exercising at least 30 min per day; SMBG; foot care; and not smoking cigarettes. Participants were asked to indicate the number of days they engaged in each of the self-care behaviours for the past 7 days. The greater the number of days reported for a behaviour the better the self-care. The validity and reliability of the SDSCA have been found to be acceptable with both European and African American diabetes patients [24, 25]. The SDSCA has demonstrated adequate test-retest reliability and evidence of validity and sensitivity to change in a number of studies [26–32]. Previous studies that investigated adherence to self-care behaviours using the SDSCA did not report not smoking cigarette as a self-care behaviour [25, 33]. Hence, not smoking cigarette was not reported as a self-care behaviour but as a demographic factor.

*BMI:* Weight was measured without shoes and wearing light clothing to the nearest kilogram using the United Nations Children’s Fund (UNICEF) electronic scale manufactured by Seca. Height was measured without shoes to the nearest centimetre using a wall-mounted standardized microtoise manufactured by Seca. BMI was calculated as body weight in kilograms divided by the squared value of body height in meters (kg/m^2^) and categorized into underweight (BMI ≤ 18.5Kg/m^2^), normal weight (18.5–24.9 Kg/m^2^), overweight (25.0–29.9 Kg/m^2^) and obese (≥30 Kg/m^2^) based on the World Health Organization (WHO) criteria [34].

*Waist circumference (WC):* was measured midway between the inferior angle of the ribs and the suprailiac crest [35] to the nearest 1 cm using a non-stretchable fibre-glass measuring tape (Butterfly, China). Participants stood in an upright position, with arms relaxed at the side, feet evenly spread apart and body weight evenly distributed in accordance with the WHO expert consultation report on waist circumference and waist-to-hip ratio [35]. Abdominal obesity was determined as a waist circumference >102 cm in men and >88 cm in women according to the WHO cut-off points and risk of metabolic complications for waist circumference [35].

*Demographic characteristics:* Age (continuous), gender, duration of diabetes since diagnosis (years), family history (yes/no), educational status (years), marital status (married and not married) and religious status (Christianity, Islamic and African traditional religion) were self-reported.

### Statistical analysis

All data were analysed using IBM SPSS version 20.0. Means and standard deviations were used to describe all continuous variables including self-care behaviours, age, duration of diabetes since diagnosis, number of cigarettes smoked per day, weight, and height. Categorical variables were reported as frequencies and percentages. Univariate associations were examined between demographic or anthropometric variables, and self-care behaviours using independent t-tests (for categorical variables) and Pearson correlations (for continuous variables).

To identify factors associated with self-care behaviours, variables that were significant in the univariate associations were entered as independent variables into stepwise regression models using forward selection. Each of the four self-care behavior scores (i.e. diet, exercise, SMBG and foot care) were included as dependent variables in separate regression models (i.e. four models in total). In all statistical analysis, a *p* < 0.05 was considered significant.

## Results

### Demographic and anthropometric variables

Of 201 patients approached, 190 (95%) consented, however, only 187 (98%) contained sufficient data for inclusion in the analysis. The demographic characteristics and anthropometric measurements of the sample are presented in Table 1. The majority of participants were female, older than 50 years, married, and followed the Islamic religion. One-third reported having diabetes for over 5 years and 38.5% had a family history of diabetes. Only three participants reported smoking cigarettes.

**Table 1:** Participant demographic and anthropometric characteristics (*n* = 187)

Gender	Frequency (%)
Male	52 (27.8%)
Female	135 (72.2%)
Mean ± SD age (years)	54.83 ± 13.32
≤ 50 years	69 (36.9%)
> 50 years	118 (63.1%)
Religious following	
Islam	154 (82.4%)
Christianity	32 (17.1%)
African Traditional Religion	1 (0.5%)
Mean number of years schooling	6.63 ± 7.23
≤ 12 years	140 (74.9%)
> 12 years	47 (25.1%)
Marital status	
Single	67 (32.8%)
Married	120 (64.2%)
Mean duration of diabetes (years)	5.43 ± 4.92
≤ 5 years	125 (66.8%)
> 5 years	62 (33.2%)
Family history	
Yes (Parent, brother, sister or own child)	21 (11.2%)
Yes (grandparent, aunt, uncle or first cousin (but no own parent, brother, sister or child)	51 (27.3%)
No	115 (61.5%)
Anthropometry	Frequency (%)
Mean BMI (kg/m^2^)	29.27 ± 6.87
Obese	75 (40.1%)
Overweight	61 (32.6%)
Normal weight	51 (27.3%)
Mean WC (cm)	98.60 ± 14.14
Abdominally obese	123 (65.8%)
Smoking status	
Non Smoker	184 (98.4%)
Smoker	3 (1.6%)
Mean number of cigarettes smoked per day	5.00 ± 1.00

### Adherence to self-care behaviours

Table 2 presents the mean number of days each diabetes self-care behaviour was reported as being performed during the last 7 days. It also specifies the percentage of participants that reported performing each of these behaviours daily. The most commonly performed diabetes self-care behaviour was participation in a specific exercise session (5.19 (2.24)) days per week) and the least was testing blood sugar level according to the number of times recommended by a health provider (2.12 (0.69)) days per week). Only 1 patient performed self-monitoring of blood glucose (SMBG) daily; 26 (13.9%) checked their feet daily and 18(9.6%) inspected the inside of their shoes every day.

**Table 2:** Participant frequency of adhering to self-care behaviours (*n* = 187)

Self-care behaviours (0 to 7 days)	Mean (SD)	Performed self-care behaviour daily (n, %)
General diet	4.37 (1.96)	16 (8.6%)
Follows a healthful eating plan	4.79 (2.09)	56 (29.9%)
Follows eating plan	3.94 (1.97)	16 (8.6%)
Specific diet	4.44 (1.31)	13 (7.0%)
Eats five or more servings of fruits and vegetables	3.25 (2.11)	25 (13.4%)
Eats high fat foods (red meat or full fat dairy products)	1.34 (1.62)	3 (1.6%)
Total diet score per week	4.40 (1.52)	5 (2.7%)
Exercise	4.78 (2.09)	40 (21.4%)
Participates in at least 30 min of physical activity	4.37 (2.56)	50 (26.7%)
Participates in a specific exercise session	5.19 (2.24)	62 (33.2%)
SMBG	2.15 (0.65)	1 (0.5%)
Tests blood sugar level	2.19 (0.74)	1 (0.5%)
Tests blood sugar the number of times recommended by your health care provider	2.12 (0.69)	1 (0.5%)
Foot care	2.86 (2.16)	18 (9.6%)
Checks feet	3.17 (2.40)	26 (13.9%)
Inspects inside of shoes	2.54 (2.22)	18 (9.6%)

Data are Mean (SD) or n (%). Mean refers to the average number of days participants adhered to a particular self-care behaviour in the last 7 days. SD = Standard deviation. Only the adherent cells are presented for brevity. All 187 participants completed all self-care items

### Associations between participant characteristics and self-care behaviours

#### Univariate associations among participant characteristics and self-care behaviours

Frequency of participation in a diabetes self-care behaviour defined by number of days per week was analysed according to demographic variables. Men (2.36 (1.02) days per week) reported greater mean (SD) days per week for blood glucose testing than women (2.07 (0.40) days per week), t (184) = 0.007. Age, marital status, duration of diabetes, family history, religious following, BMI (normal/overweight/obese) and WC (abdominally obese vs not) were not associated to frequency of participation in any of the self-care behaviours.

#### Multivariate associations between participant characteristics and self-care behaviours

Table 3 presents the regression models of factors associated with adherence to the four self-care behaviours. Number of years in school was associated with frequency of adhering to diet (*r* = 0.223, *p* = 0.002), exercise (*r* = 0.168, *p* = 0.022), and foot care (*r* = 0.153, *p* = 0.037). Male gender was associated with higher frequency of performing SMBG (*r* = 0.198, *p* = 0.007).

**Table 3:** Factors associated with participant frequency of adhering to self-care behaviours

Variable	F	B	SE of β	*p*-value	Partial correlation	Adjusted R^2^
Diet	6.203					0.053
Number of years in school		0.226	0.02	0.002	0.223	
Exercise	5.360					0.023
Number of years in school		0.168	0.021	0.022	0.168	
SMBG	7.484					0.034
Gender^a^		0.198	0.104	0.007	0.198	
Foot care	4.415					0.023
Number of years in school		0.153	0.022	0.037	0.153	

^a^Gender: Male = 1, Female = 0. Diet includes both specific and general diet

## Discussion

This study described-the frequency of adhering to four self-care behaviours in adult Ghanaian type 2 diabetes patients and factors associated with performing these self-care behaviours. Exercise was the most commonly performed self-care behaviour and SMBG was the least adhered to by the participants. More education and being female were associated with adherence to self-care behaviours. Given that self-care is a multidimensional concept, factors associated with each of the four self-care behaviours were investigated separately using multivariate analysis: diet; exercise; SMBG and foot care.

### Self-care behaviours

#### Exercise

The frequency of exercise reported in this study is one of the highest reported among type 2 diabetes patients. Participants performed physical activity of at least 30 min for an average of 4.37 days per week. This is higher than the 2.7 days reported among both African American type 2 diabetes patients [25] and diabetic patients from three rural Appalachian communities [33]. These findings are however consistent with those among type 2 diabetes patients in Ethiopia [36] and immigrant Filipino Americans living with diabetes [37]. The relatively high exercise adherence in this study could be due to most patients generally having to walk for transport. Despite the relatively high mean number of days of performing physical activity in this study, less than 35% of the participants exercised for 30 min daily or participated in a specified exercise session every day during the past week. Several barriers may have prevented daily adherence to physical activity including the risk of hypoglycemia; inadequate access to conducive environment and facilities to perform physical activity and fear of increasing blood pressure [37–40].

#### Diet

The frequency of following general (4.37 days per week) and specific diet (4.44 days per week) in these type 2 diabetes patients is similar to the 4.1 days per week found among a rural population of diabetes patients [33]; and 4.37 days and 4.09 days per week following general and specific diets respectively among diabetes patients with a rural background [41]. These findings are however lower than those reported among African American type 2 diabetes patients [25]; in type 2 diabetes patients from an urban setting in the US [41] and Chinese American type 2 diabetes patients [42]. The patients’ performance on specific self-care behaviours for diet were less desirable: less than 15% of them ate fruits and vegetables on a daily basis and less than one-third followed a healthy eating plan daily. Seasonality of fruits and vegetables and cost might have contributed to the low adherence to diet.

#### Self-monitoring of blood glucose

Decreased ability to adjust medication dosages, dietary intake and physical activity could arise, if SMBG is not performed as recommended [37]. Despite this, SMBG was the least performed self-care behaviour in these Ghanaian type 2 diabetes patients with only 1 patient doing SMBG daily. This is among the lowest frequency of performing SMBG among type 2 diabetes patients in sub-Saharan Africa and other parts of the world. In type 2 diabetes patients in Harari, Eastern Ethiopia, 2.6% of a sample of type 2 diabetes patients performed SMBG daily [43]. African American diabetes patients reported an average of 4.7 days per week of performing SMBG in a cross-sectional study in the US [25]. Furthermore, a study of type 2 diabetes in low-income urban Puerto Ricans in the US, found that 60% of the participants performed SMBG once or twice daily [44]. The current findings are only similar to those reported among rural diabetes patients in the US in which participants reported an average of 2.15 days per week of performing SMBG [33]. Inadequate access to glucose monitoring machines, cost of test strips and needles, lack of requisite knowledge and skills to perform and interpret SMBG readings; lack of provider support; fear of testing and pain and preference for traditional and alternative medicine [45–49] may be responsible for the low SMBG in Ghanaian type 2 diabetes patients.

#### Foot care

Participants’ frequency of checking their feet in the last 7 days was lower than the 4.33 [41] and 4.8 days [25] per week, and the 42.1% who checked their feet per day [42] reported in previous research. Similar low levels of foot care practices have been reported previously [16, 50] in Sub-Saharan diabetes patients. Several factors could contribute to the low practice of foot care in these type 2 diabetes population including lack of knowledge on how to perform foot care [16, 50]; poor provider-patient communication; inconvenience for work; and poverty affecting patient’s inability to purchase appropriate footwear [16].

### Factors associated with self-care behaviours

The study results suggest that number of years of education plays an important role in diabetes self-care behaviours such as diet, exercise and foot care. These findings concur with those of previous studies reported among type 2 diabetes patients in Sub-Saharan Africa [36, 50] and from China [42, 51]. Patients with more years of education may be more likely to comprehend recommended self-care behaviours than their less educated counterparts because they may be able to read and become more informed of the benefits of adherence. This is a cause of concern since the majority of the Ghanaian adults with type 2 diabetes and those reported previously are usually less educated [22, 52, 53]. Unavailability of linguistically and culturally relevant diabetes self-care education resources in the Ghanaian setting, as well as patients’ inability to interact with healthcare providers due to low literacy may be some of the factors that makes it difficult for effective counselling on self-care behaviours. Inadequate awareness of health concepts may also be another contributing factor.

It was also found that gender plays an important role in SMBG in that men were more likely than women to perform SMBG. A qualitative study from Canada [54] reported that while women were more concerned with their fears and anxieties, men focused on the technical aspects of SMBG and were more likely to experiment with SMBG. Thus, women may have less confidence to use glucometers resulting in their reluctance to perform SMBG. Furthermore, women also receive less family support for self-care; may lack confidence; may self-blame themselves more for the condition and may also allow the needs of children and spouses to take precedence over their needs [25, 55, 56]. It is imperative that providers are aware of the gendered dimensions in diabetes self-care and address these in their counselling sessions with patients [55].

### Association between adherence to self-care behaviours and body weight

Contrary to the findings of Dixon et al. [23] in Australia, BMI and WC were not significantly associated to any of the self-care behaviours investigated in this study. The diabetes populations of the Dixon et al study and the current study could differ in their perception of the health risks of excess body weight. Excess body weight is generally considered as a sign of beauty, affluence and well-being in most parts of sub-Saharan African countries including Ghana [57, 58] but generally considered a health risk in many developed countries including Australia. Thus the diabetes population of the current study might be less concerned about their body weight and less likely to adopt strategies to control it.

### Strengths and Limitations

This is the first study to investigate more than one self-care behaviour in type 2 diabetes patients in Ghana. Another strength of the study is the use of a reliable, valid and widely used instrument for the assessment of self-care behaviours in diabetes patients. The main limitations of the study are the cross-sectional design, which cannot establish causality, and use of self-report to measure adherence, making it liable to social desirability bias. Although social desirability bias might have occurred, the self-reported self-care behaviours were, in general, low. In addition, our findings regarding SMBG would have been easier to interpret if we had collected information as to how many of our type 2 diabetes patients owned glucometers. Women and those with less education appeared to be over-represented in the sample, which may limit the generalizability of the results. However, our diabetes patient population had similar characteristics with diabetes populations reported in studies from other parts of Ghana [22, 59].

### Implications and future research

Given the link between self-care behaviours and health outcomes of diabetes patients, the low adherence found in this study is a concern. Effective strategies are needed to help improve the diabetes self-care behaviours of adult Ghanaian type 2 diabetes patients. Our findings could be relevant to the type 2 diabetes patient population of several developing countries with challenged health systems like Ghana. Future research should explore both patient and provider barriers to performing effective self-care behaviours in diabetes patients. Such data will inform the design of tailored interventions to improve adherence to self-care behaviours. There is also a need to conduct more research about how to effectively communicate about self-care behaviour with populations who have low literacy or health literacy. In addition future studies should explore the effect of performing recommended self-care behaviours on clinical outcomes of diabetes patients in the Ghanaian setting.

## Conclusion

This study has shown that the performance of self-care behaviours, SMBG and foot care in particular, are sub-optimal among Ghanaian adults with type 2 diabetes. The sociodemographic factors associated with poor adherence were lower levels of education and female gender. Further research to identify the barriers to effective self-care behaviours, particularly among those with a lower educational level and women, is warranted.

## Abbreviations

BMI: Body mass index
SDSCA: Summary of diabetes self-care activities measure
SMBG: Self-monitoring of blood glucose
SPSS: Statistical Program for the Social Sciences
UNICEF: United Nations Children’s Fund
WC: Waist circumference
WHO: World Health Organization
